# Comparison and screening of chemical topping agents for optimizing root-shoot coordination, photosynthetic efficiency and yield in south of the Tianshan mountains in Xinjiang

**DOI:** 10.3389/fpls.2026.1795237

**Published:** 2026-04-20

**Authors:** Yu Xiao, Xiaofeng Wang, Huqiang Li, Jiao Lin, Qiang Hu, Hongqiang Dong, Zhiguo Zhou, Mingwei Du, Nan Cao, Sumei Wan

**Affiliations:** 1 College of Agriculture, Tarim University, Alar, China; 2 Key Laboratory of Genetic Improvement and Efficient Production for Specialty Crops in Arid Southern Xinjiang of Xinjiang Corps, Tarim University, Alar, China; 3 College of Agriculture, Nanjing Agricultural University, Nanjing, China; 4 College of Agronomy and Biotechnology, Zhongguo Agricultural University, Beijing, China

**Keywords:** chemical topping, fiber quality, photosynthetic parameters, root productivity, yield

## Abstract

**Background:**

Although chemical topping agents are now widely used in cotton production, there is a lack of research on screening specialized formulations tailored to the unique ecological conditions of cotton region south of the Tianshan Mountains in Xinjiang. Current reliance on a single-agent application regime often leads to suboptimal efficacy. Given that the root-shoot system is a core determinant of high cotton yields, the underlying mechanism by which chemical topping agents modulate root-shoot coordination remains largely elusive.

**Methods:**

A two-year field trial was conducted during 2024 and 2025, involving three different chemical topping agents (X1: zifengding agent, X2: paclobutrazol-copper complex, X3: paclobutrazol-zinc complex), manual topping (CK) was conducted as a control. A total of four treatments were included.

**Results:**

Different chemical topping treatments effectively controlled plant height (PH) and increased number of fruiting branches (NFB) compared to manual topping (CK). It significantly enhanced the net photosynthetic rate (Pn), stomatal conductance (Gs), and transpiration rate (Tr), while reducing intercellular CO_2_ concentration (Ci). Compared with the CK treatment, the X1, X2, and X3 treatments increased the boll capacity of the root system(BCR) by 9.40%-9.60%, 15.60%-17.50%, and 20.00%-22.40%, and increased the boll loading of the root system (BLR) by 3.30%-3.80%, 9.80%-10.20%, and 12.40%-14.10%, respectively. Consequently, biomass accumulation peaked under X3 treatment, increasing seed cotton and lint yields by 10.44%-12.17% and 9.20%-11.00%, respectively, while also improving fiber strength.

**Conclusion:**

This study breaks the extensive “one-size-fits-all” application mode. Specifically, the X3 treatment enables precise and localized application of chemical topping technology, providing theoretical and technical support for high-yield, high-quality, efficient, and green cotton cultivation.

## 1 Introduction

Cotton is one of the most important economic crops in Xinjiang, accounting for more than 80% of China’s total planting area and output, serving as a pillar industry that boosts farmers’ income and prosperity and drives regional economic development (Yang et al., 2022). Under intensive cultivation conditions, cotton plants exhibit excessive vegetative growth and canopy closure, resulting in the deterioration of the canopy light environment, increased boll and square abscission, and ultimately decreased seed cotton yield (Huang et al., 2025). Topping, as a key agronomic practice in cotton production, can effectively inhibit apical dominance, reduce the formation of non-effective squares, and promote the coordination of vegetative and reproductive growth, which serves as an important regulatory measure to achieve high yield and superior quality in cotton (Qi et al., 2023). However, traditional manual topping is characterized by high labor intensity and elevated production costs, making it challenging to meet the demands of cotton production (Adeleke, 2024). Consequently, there is an urgent need for efficient alternatives suitable for large-scale mechanized farming. With advancements in agricultural technology, chemical topping has emerged and gradually replaced manual topping (Xu et al., 2023).

In recent years, with the continuous and widespread application of chemical topping technology, related research has gradually expanded (Zhang et al., 2024). Studies have demonstrated that chemical topping agents could inhibit the apical growth of cotton plants and significantly increase the number of fruiting branches and bolls, thus increasing cotton yield (Zhu et al., 2022). Wu et al. (2023) reported that the number of fruiting branches in cotton plants under chemical topping treatments increased by 1.40-1.60 compared with manual topping, with both boll number per plant and seed cotton yield being higher than manual topping. Similarly, Wang et al. (2024) found that chemical topping induced significant morphological changes in cotton, with a 13.20% increase in plant height and a 2.30% enlargement in stem diameter internode relative to manual topping. This advantage of chemical topping exerts a positive promoting effect on the growth and development of cotton plants, which not only boosts cotton yield but also optimizes yield components.

As the core organ responsible for water and nutrient uptake (Li et al., 2025b), the root productivity directly determines the capacity for nutrient acquisition, modulates the shoot source-sink relationship, and thereby provides the material foundation for photosynthesis (Qin et al., 2023). Through photosynthesis, plants convert assimilated resources into dry matter, ultimately modulating yield formation (Simkin et al., 2019). Consequently, improvements in both root productivity and photosynthetic efficiency are considered key physiological mechanisms through which chemical topping enhances cotton yield. Tian et al. (2022) reported that under the same drip irrigation rate, moderate concentrations of chemical topping agents significantly increased the net photosynthetic rate, stomatal conductance, and transpiration rate by 4.00%-7.20%, 6.80%-17.10%, and 5.20%-17.60%, respectively, from the full flowering stage to the boll opening stage. Reproductive organ biomass accumulated rapidly, with seed cotton yield increasing by 6.60%-11.80% compared with manual topping. At 40 days after topping, the average net photosynthetic rate, stomatal conductance, and transpiration rate under chemical topping treatments increased by an average of 37.50%, and seed cotton yield was 3.60%-11.10% and 1.60%-10.90% higher than manual topping, respectively (Shi et al., 2024). Collectively, these findings indicate that chemical topping agents enhance leaf gas exchange capacity and photosynthetic efficiency, thereby promoting the accumulation and partitioning of dry matter, and thus effectively improving cotton yield.

As a triazole plant growth regulator, paclobutrazol inhibits gibberellin biosynthesis, suppresses cell division and elongation in the apical meristem of cotton, and promotes reproductive growth, which has been widely applied for chemical topping and plant type regulation in cotton production (Temiz et al., 2009). Studies have found that the synergistic application of paclobutrazol with metal ions can enhance the efficacy of chemical topping and optimize plant type in cotton, resulting in increases of approximately 5.80% and 4.50% in seed cotton yield and lint yield, respectively, compared with manual topping (Shen et al., 2025). In addition, triazole metal complexes are promising to improve its bioactivity and reduce environmental toxicity (Park et al., 2022). However, studies on its effects on cotton root productivity and the interrelationships among growth, photosynthetic characteristics, root productivity, growth, seed cotton yield and fiber quality remain relatively poor understood. Therefore, this study was conducted with manual topping as the control to investigate the effects of three chemical topping agents: zifengding agent (X1), paclobutrazol-copper complex (X2), and paclobutrazol-zinc complex (X3) on cotton plants, screen out the optimal chemical topping agent, and provide a theoretical basis for the application and popularization of chemical topping agents in cotton region south of the Tianshan Mountains in Xinjiang. Furthermore, this study also explored the inherent mechanism by which different chemical topping agents affect cotton plant growth, development, and yield formation by regulating the coordination of root-shoot growth and source-sink relationship in cotton.

## 2 Materials and methods

### 2.1 Experimental site and design

**Figure 1:**
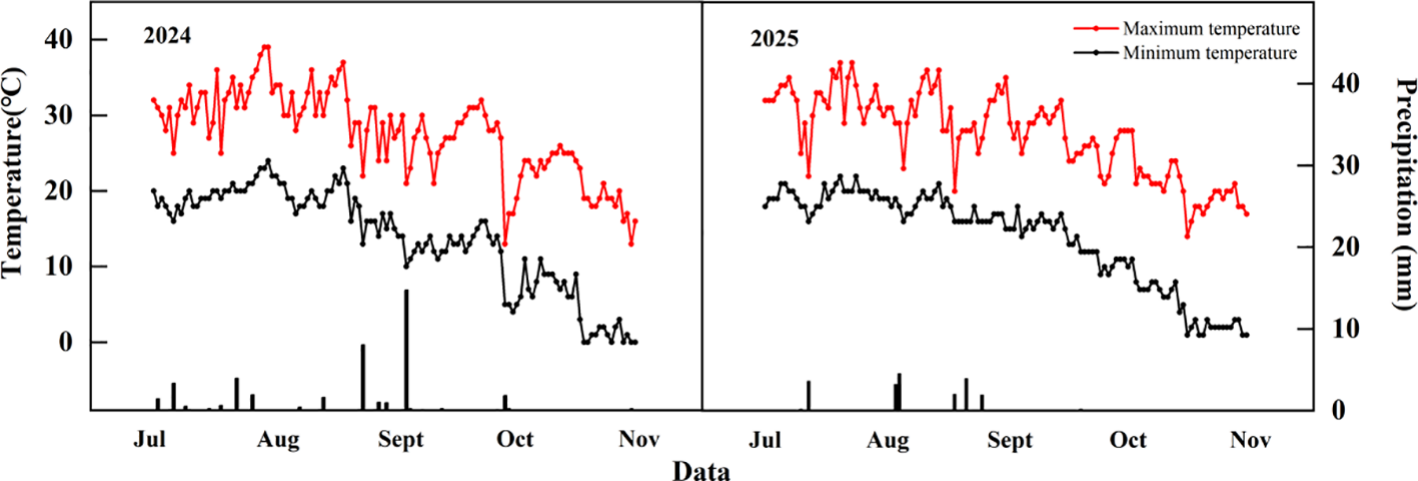
Temperature and precipitation after the peak of the test area in 2024 and 2025.

The field experiment was conducted from 2024 to 2025 at the Modern Agricultural Training Base for Industry-Education Integration in Southern Xinjiang (81°50’E, 40°39’N), Tarim University, Alar City, Xinjiang. This region has a warm temperate continental arid climate. The soil is classified as sand according to the United States Department of Agriculture (USDA) soil taxonomy, with a soil pH of 7.74, an available phosphorus (P) content of 17.60 mg kg^-1^, and an available potassium (K) content of 108.30 mg kg^-1^. The climatic conditions of the experimental site are shown in **Figure 1**.

The field experiment was laid out in a randomized complete block design. With four treatments established, manual topping (the terminal bud was removed at the one leaf and one heart stage, CK) was set as the control, and the treatments included zifengding agent (X1) was provided by Hebei Guoxin Nuonong Biotechnology Co., Ltd. (Hebei, China), with an application rate of 225 g ha^-1^ for the agent, 150 g ha^-1^ for the adjuvant, paclobutrazol-copper Complex (X2) was applied at 120 g ha^-1^, and paclobutrazol-zinc Complex (X3) was also applied at 120 g ha^-1^ (Shen et al., 2025). Chemical topping treatments were manually applied in the field using a backpack sprayer. Each treatment was replicated three times, with each plot having an area of 165.60 m^2^ (9.20 m×18.00 m). The randomization of plots was implemented to account for spatial variability in soil properties and minimize environmental bias.

Tahe No.2 and Xintamian No.7 were used as the test varieties in 2024 and 2025, respectively. Both Tahe No.2 and Xintamian No.7 are early-to-medium maturing non-transgenic conventional upland cotton varieties independently bred by Xinjiang Tarim River Seed Industry Co., Ltd. They belong to the same series from the same breeding institution but represent different generations. Seeds were sown on April 19, 2024 and April 20, 2025, and topping was conducted on July 9 (2024) and July 11 (2025), respectively. Sowing was performed using a precision hole seeder with punching on plastic film for cotton, with one seed per hole and a sowing depth of 2.50-3.00 cm to guarantee one plant per hill. The planting pattern was six rows per plastic film (66 cm+10 cm), with a planting density of 220,000 plants ha^-1^ and a plant spacing of 10.50 cm. Management was conducted in accordance with the local conventional under-film drip irrigation planting pattern. A total of 8 irrigations were applied during the cotton growing period, with an irrigation interval of 6–9 days. The N fertilizer (urea, 46% N) was applied at 420 kg ha^-1^, phosphorus fertilizer (P_2_O_5_, 46%) at 185 kg ha^-1^, and potassium fertilizer (K_2_O, 52%) at 120 kg ha^-1^. All other field management practices were consistent with those adopted for field-grown cotton in this region.

### 2.2 Plant sampling and analysis

Before cotton topping, five uniform and representative cotton plants with consistent growth vigor were randomly selected from each plot and tagged with labels for identification. Agronomic traits were measured at 0, 7, 14, 21, and 28 days after topping (DAT), including plant height, stem diameter, and number of fruit branches of cotton were investigated. The average value of the replicated data was calculated for each index. Plant height (PH) was measured from the cotyledon node to the apex using a tape measure. Stem diameter (SD) at the midpoint between the cotyledon and first true leaf nodes was recorded with an electronic Vernier caliper (± 0.01 mm).

At 0, 7, 14, 21, and 28 DAT, five cotton plants were randomly sampled from each treatment. A soil block of 40 cm × 10 cm × 60 cm was excavated around each sampled plant, and the whole plant including the root system was extracted as intact as possible. The samples were then divided into different organs: root, stem, leaf, flower, and boll. Collected roots were washed gently with running water over a 0.25 mm sieve to eliminate soil residues. All plant materials were first oven-dried at 105 °C for 30 min, followed by drying at 80 °C for 48 h to a constant weight, after which they were weighed.

### 2.3 Cotton photosynthetic parameters

Photosynthetic parameters were measured at 7, 14, 21, and 28 DAT. The net photosynthetic rate (Pn), stomatal conductance (Gs), intercellular CO_2_ concentration (Ci), and transpiration rate (Tr) of the functional leaves on the cotton plant main stem were determined using a LI-6400 Portable Photosynthesis System (Li-COR, Lincoln, NE, USA). Measurements were conducted on the third fully expanded leaf from the top under clear weather conditions between 11:00 and 14:00. Each leaf was measured three times, and the average value was calculated.

### 2.4 Cotton root productivity

The productive capacity of cotton roots can be quantified using two key metrics: boll loading of the root system (BLR) and boll capacity of the root system (BCR) (Li et al., 2025a).

(1) $$BLR\;\left(boll\;g^{-1}\right)\;=\;Boll\;number\;\left(boll\;ha^{-1}\right)/Root\;biomass\;\left(kg\;ha^{-1}\right)\;\times \;1000$$

(2) $$BCR\;\left(g\;g^{-1}\right)\;=\;Boll\;biomass\;\left(kg\;ha^{-1}\right)/Root\;biomass\;\left(kg\;ha^{-1}\right)\;\times \;1000$$

The root-shoot ratio (RSR) was calculated as:

(3) $$RSR\;=\;Root\;biomass\;\left(kg\;ha^{-1}\right)/Shoot\;biomass\;\left(kg\;ha^{-1}\right)$$

### 2.5 Cotton biomass

The biomass of each organ of cotton plants was measured at 0, 7, 14, 21, and 28 DAT. Three representative cotton plants with intact root systems were excavated from each plot, and separated into roots, stems, leaves, and buds, flowers, and bolls for sample preparation. The samples in oven at 105 °C for 30 minutes, and then the oven temperature was reduced to 80 °C until the samples were dried to a constant weight. The biomass was calculated based on the weighed values of the dried samples.

### 2.6 Cotton yield and cotton fiber quality

During the boll opening stage, a 6.67 m^2^ sampling area with uniform growth vigor was selected from each plot for yield determination. The plant number, total boll number, young bolls (diameter ≤ 2 cm), mature bolls (diameter > 2 cm), and rotten bolls were investigated. From each plot, 100 open bolls were harvested to determine seed cotton weight and single boll weight. After harvesting, the cottonseed and fiber were separated using a cottonseed delinter (MBRJ-33, Hebei, China). In this experiment, all measurements were completed with the use of the PREMIER ART 3 fully automatic large capacity cotton fiber quality detection system (origin: Jiangsu, China) to ensure the accuracy and reliability of cotton fiber quality measurement. Prior to the measurement, the instrument was calibrated as instructed.

### 2.7 Statistical analysis

Statistical analyses were performed using SPSS Statistics 22.0 software (IBM Corp., Armonk, NY, USA). One-way analysis of variance (ANOVA) was conducted, followed by Duncan’s multiple range test to determine significant differences at the P< 0.05 level. All figures were plotted using Origin 2024 software (OriginLab Corp., Northampton, MA, USA). Partial least squares path modeling (PLS–PM) was conducted using the “plspm” package in R Studio to evaluate the direct and indirect effects of the three agents on agronomic traits, photosynthetic parameters, root productivity, biomass, and yield. Correlation analysis of the photosynthetic parameters, root productivity, biomass, and yield was carried out in Origin 2024 with the correlation plot app.

## 3 Results

### 3.1 Cotton agronomic traits

Results from the two-year field trials demonstrated that different chemical topping agents exerted significantly effects on cotton PH, SD, and NFB. No significant differences were observed among all treatments at 0 DAT. Differences among treatments gradually emerged from 7 DAT onwards and had the maximum level in 28 DAT (**Table 1**).

**Table 1:** The dynamic changes of cotton agronomic traits under different chemical topping agents during 2024-2025.

DAT	Treatment	2024			2025		
		PH (cm)	SD (mm)	NFB (No)	PH (cm)	SD (mm)	NFB (No)
0 d	CK	62.57 a	10.34 a	9.46 a	69.63 a	10.11 a	9.63 a
	X1	63.90 a	9.85 a	9.39 a	69.57 a	9.95 a	9.30 a
	X2	62.86 a	9.44 a	9.22 a	69.97 a	10.07 a	9.33 a
	X3	63.62 a	9.27 a	9.42 a	68.63 a	9.85 a	9.97 a
7 d	CK	66.34 b	10.77 a	9.37 b	73.40 a	10.48 a	10.07 a
	X1	72.31 a	10.39 a	10.78 a	75.03 a	10.30 a	10.40 a
	X2	72.26 a	9.71 a	10.14 ab	74.90 a	10.21 a	10.27 a
	X3	69.42 ab	9.90 a	9.75 ab	75.20 a	10.41 a	10.33 a
14 d	CK	69.23 b	12.11 a	9.69 b	74.73 b	11.34 a	10.40 b
	X1	78.99 a	11.02 ab	11.22 a	83.20 a	10.83 b	11.10 a
	X2	78.11 a	10.26 b	10.78 ab	82.37 a	10.98 b	11.00 a
	X3	73.37 b	10.51 b	10.11 ab	82.57 a	11.17 ab	10.80 ab
21 d	CK	72.64 c	12.49 a	9.97 b	78.60 b	12.70 a	10.53 b
	X1	84.84 a	11.57 ab	11.71 a	89.40 a	11.65 b	11.73 a
	X2	83.42 a	10.87 b	11.06 ab	88.00 a	12.12 ab	11.63 a
	X3	78.67 b	10.91 b	10.56 ab	87.00 a	12.28 ab	11.26 ab
28 d	CK	74.42 c	13.15 a	10.20 b	81.60 b	13.19 a	10.60 b
	X1	87.09 a	12.20 ab	12.06 a	94.20 a	12.23 c	12.20 a
	X2	86.81 a	11.18 b	11.22 ab	94.11 a	12.42 bc	11.93 a
	X3	80.84 b	11.85 ab	10.89 ab	91.97 a	12.67 ab	11.67 a

DAT, days after topping; PH, plant height; SD, stem diameter; NFB, number of fruiting branches; CK, manual topping; X1, zifengding agent; X2, paclobutrazol-copper complex; X3, paclobutrazol-zinc complex. Different lowercase letters indicate significant differences between treatments at the 5% probability level (p< 0.05).

The cotton plants of X1 treatment had the highest PH across two years, with an increase of 15.44%-17.02% compared with CK treatment in 28 DAT. In contrast, X2 and X3 treatments significantly decreased the cotton PH relative to X1 treatment. For SD, the CK treatment maintained the highest in two years, while the minimum value was recorded in the X2 treatment in 2024 and the X1 treatment in 2025, representing a reduction of 7.28% and 14.98%, respectively, compared with CK treatment. For NFB, the X1 treatment had the highest value, and no significant difference was detected among X1, X2, and X3 treatments. Compared with CK treatment, the X1, X2, and X3 treatments increased the NFB by 15.09%-18.24%, 10.00%-12.55%, and 6.76%-10.09%, respectively, in 2024 - 2025.

### 3.2 Photosynthetic rate

**Figure 2:**
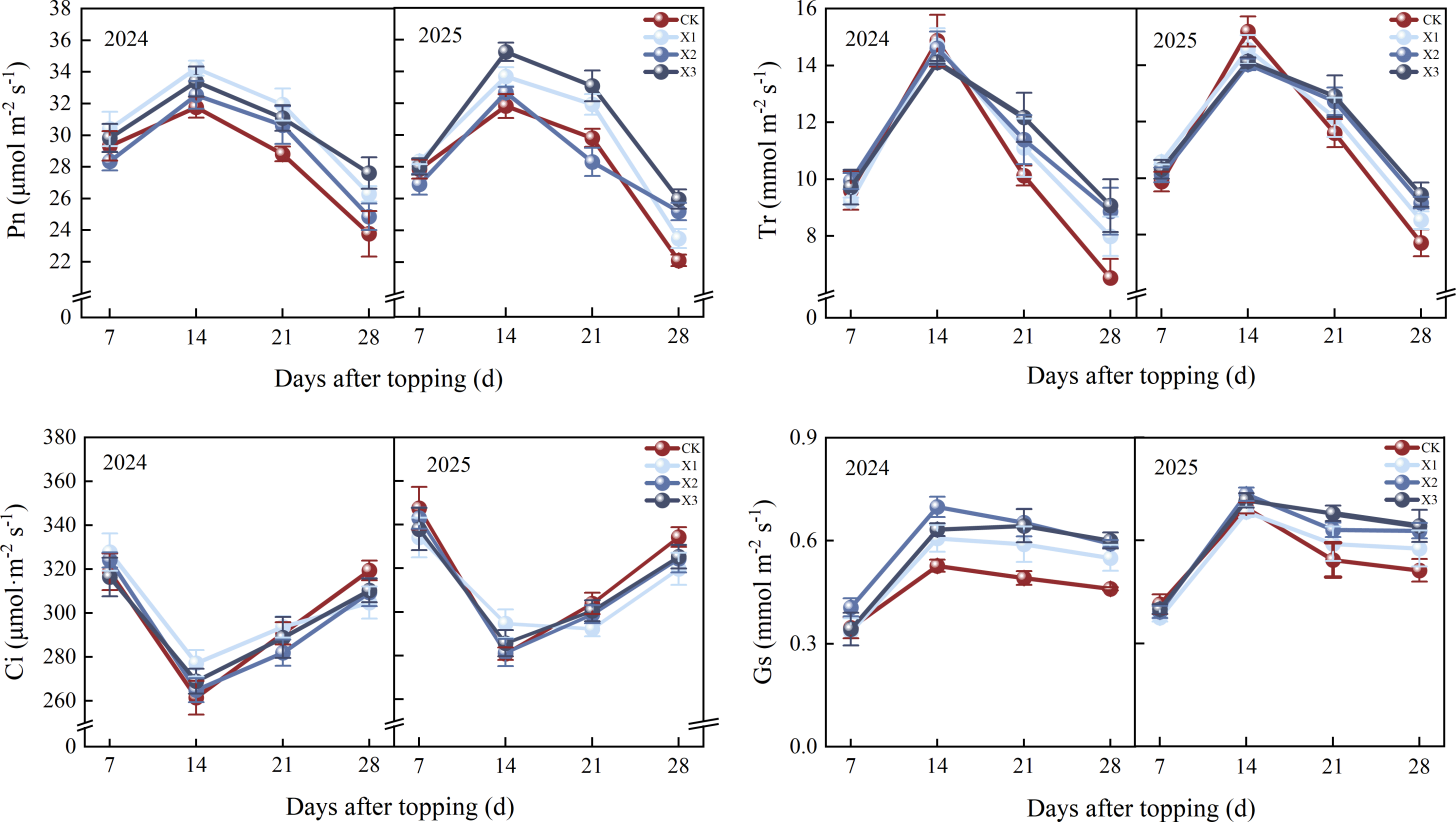
Effects of different chemical topping agents on net PN, photosynthetic rate; Tr, transpiration rate; Ci, intercellular CO_2_ concentration, and Gs, stomatal conductance of cotton from 2024-2025; CK, manual topping; X1, zifengding agent; X2, paclobutrazol-copper complex; X3, paclobutrazol-zinc complex. Different lowercase letters indicate significant differences between treatments at the 5% probability level (*p*< 0.05).

The results indicated that the Pn of cotton first increased and then decreased as the DAT increased (**Figure 2**), reaching peak at 14 DAT, all chemical topping treatments showed higher Pn than CK treatment, with the X1 and X3 treatments increasing Pn by 7.73% and 10.70% compared with CK treatment in 2024 and 2025, respectively. Differences among treatments were maximized in 28 DAT, with the X3 treatment exhibiting the highest Pn, ranging from 25.96-27.61 μmol CO_2_ m^-2^ s^-1^, which corresponded to a 16.15%-17.47% increased relative to CK treatment.

The two-year experimental results demonstrated that different chemical topping agents exerted a significant effect on the Tr of cotton leaves, and its dynamic variation first increased and then decreased. The Tr of all treatments reach peaked at 14 DAT, and then decreased over time. The X3 treatment had the highest Tr at 28 DAT across the two years, ranging from 9.07-9.41 mmol m^-2^ s^-1^, which was 28.00%-38.90% higher than CK treatment.

The Ci in cotton leaves exhibited a decreased and then increased trend as DAT increased, with consistent variation patterns observed in 2024 and 2025. The Ci of all treatments had the minimum value at 14 DAT, followed by a gradual increase thereafter. The Ci values of all chemical topping treatments were lower than that of CK treatment in 28 DAT. Compared with CK treatment, the X1, X2, and X3 treatments reduced Ci by 1.39%-4.37%, 0.94%-2.97%, and 2.51%-2.71%, respectively, in 2024-2025.

In contrast, the Gs of cotton leaves also exhibited increased and then decreased as DAT increased, with consistent variation trends observed in 2024 and 2025. The Gs of all treatments peaked at 14 DAT, and then decreased over time. The Gs of all chemical topping treatments were higher than those of CK treatment in 28 DAT, the X3 treatment achieved the highest Gs value in 2024 - 2025, ranging from 0.60 to 0.64 mmol m^-2^ s^-1^, which were 60.42%-75.42% higher than those of CK treatment.

### 3.3 Cotton root productivity

**Figure 3:**
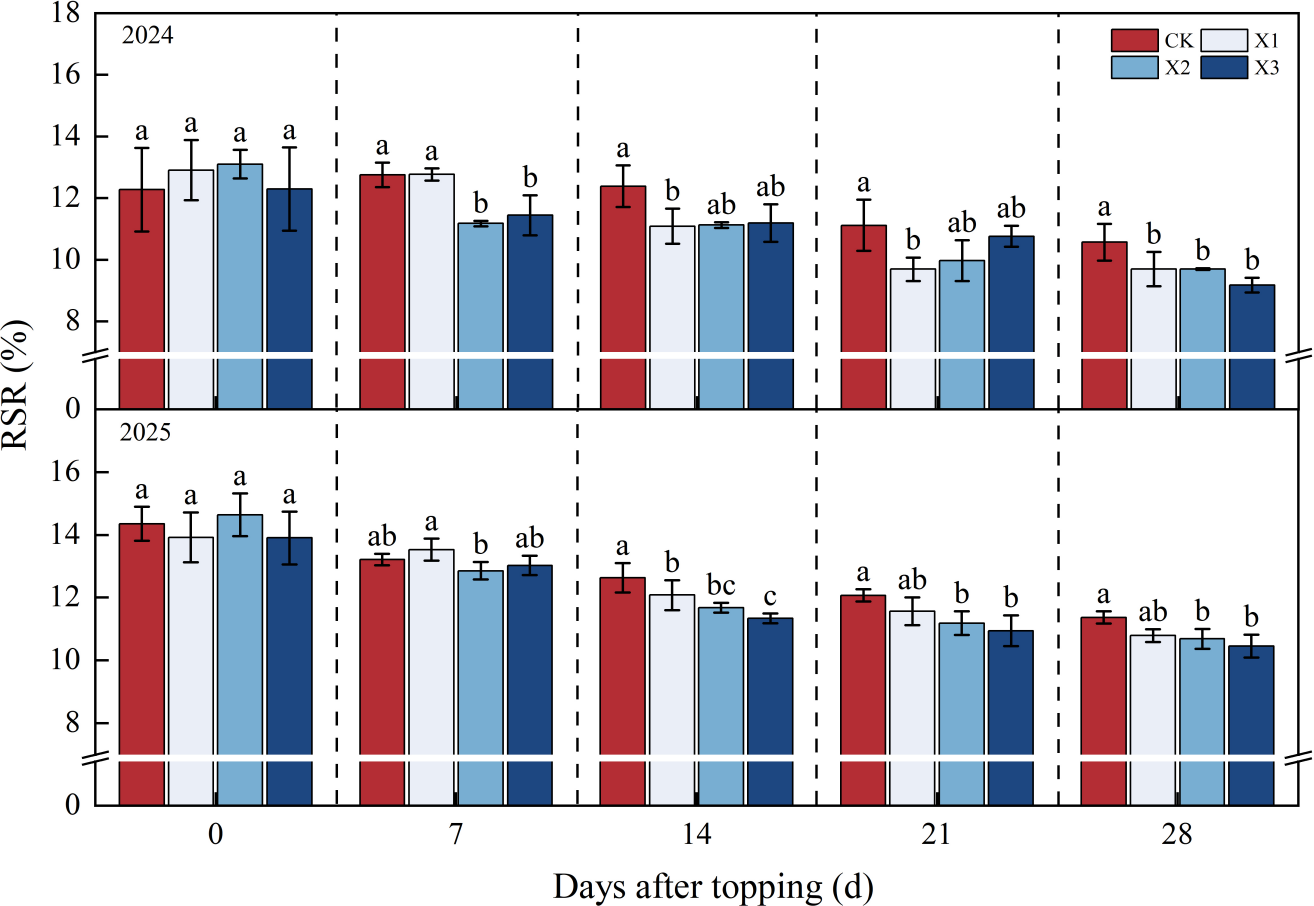
Effects of different chemical topping agents on root-shoot ratio in 2024-2025. BSR, root-shoot ratio; CK, manual topping; X1, zifengding agent; X2, paclobutrazol-copper complex; X3, paclobutrazol-zinc complex. Different lowercase letters indicate significant differences between treatments at the 5% probability level (*p*< 0.05).

The root-shoot ratio (RSR), boll loading of the root system (BLR), and boll capacity of the root system (BCR) as shown in Equations 1–3. In the two-year experiment, the RSR of all treatments exhibited a gradual downward trend as the number of DAT increased (**Figure 3**). At 28 DAT, compared with CK treatment, the RSR of X2 and X3 treatments were significantly decreased by 5.99%-8.26% and 3.3%-8.27%, respectively. However, there was no significant difference between X2 and X3 treatments across the two experimental years.

**Figure 4:**
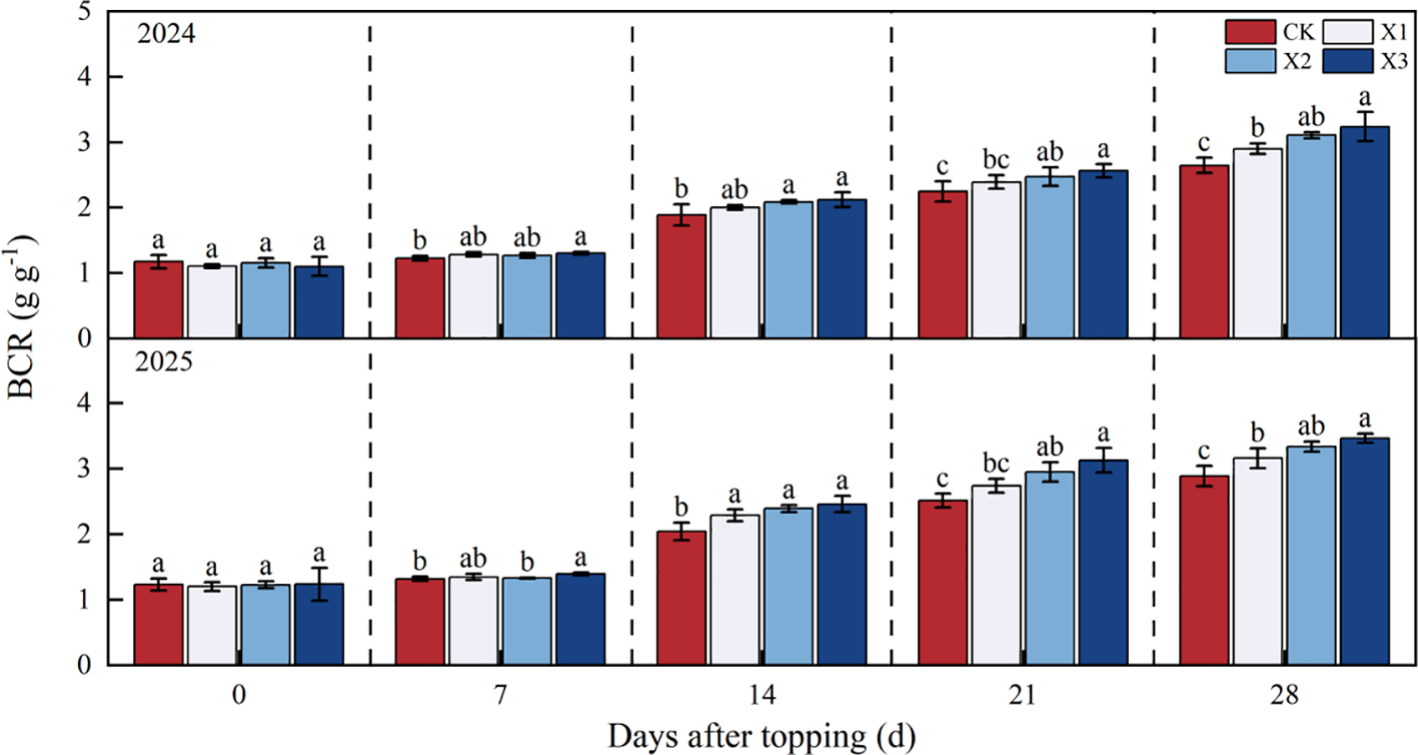
Effects of different chemical topping agents on boll capacity of the root system in 2024-2025. BCR, boll capacity of the root system; CK, manual topping; X1, zifengding agent; X2, paclobutrazol-copper complex; X3, paclobutrazol-zinc complex. Different lowercase letters indicate significant differences between treatments at the 5% probability level (*p*< 0.05).

The BCR showed a continuous increasing trend as DAT increased, with consistent variation patterns observed over the two years (**Figure 4**). The BCR of all chemical topping treatments were higher than those of CK treatment in 28 DAT, the X3 treatment achieved the highest BCR value, compared with CK treatment, the X1, X2, and X3 treatments increased the BCR by 9.40%-9.60%, 15.60%-17.50%, and 20.00%-22.40%, respectively, in 2024 - 2025. However, no statistically significant difference was observed between X2 and X3 treatments over the two years.

**Figure 5:**
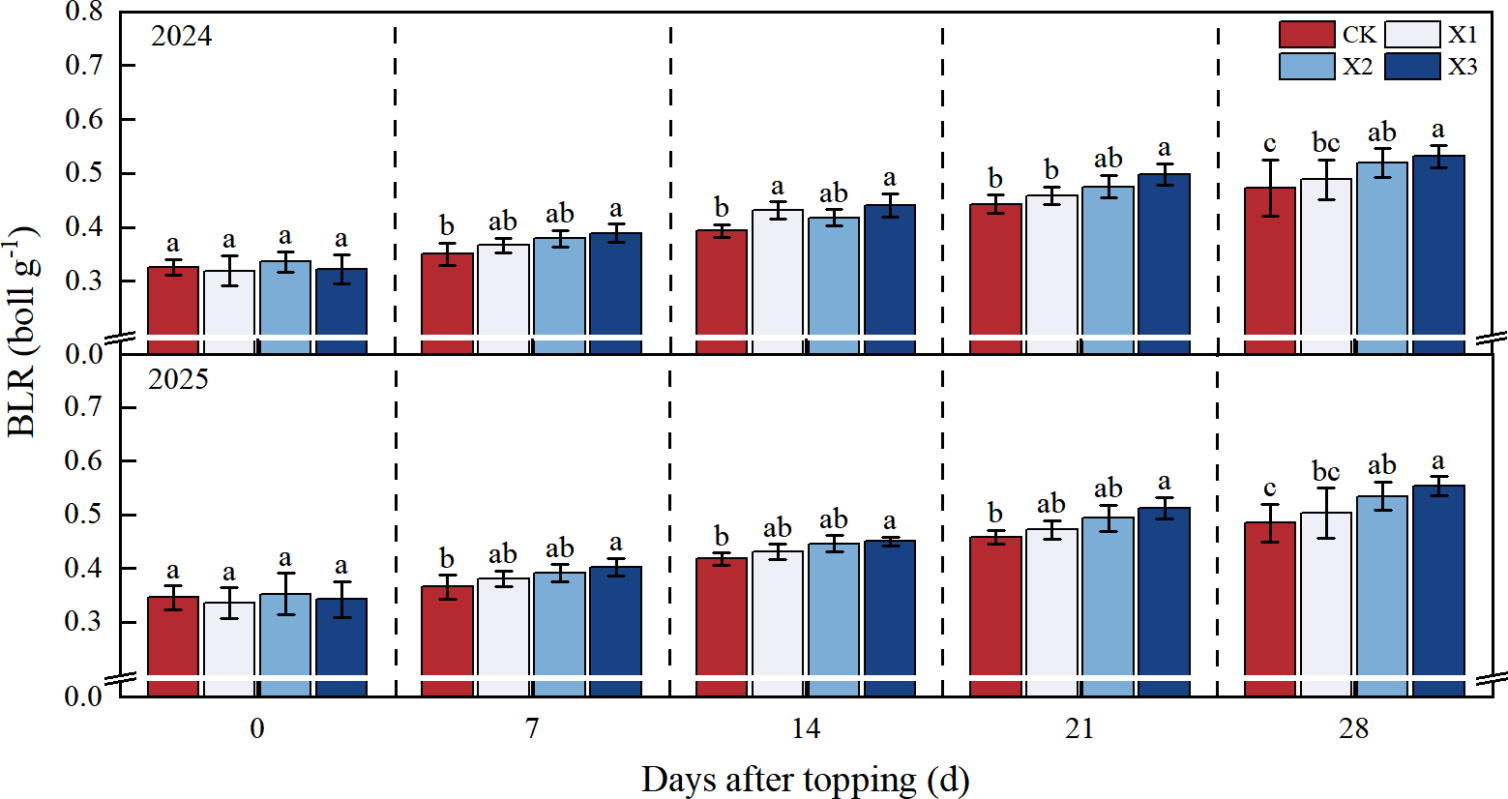
Effects of different chemical topping agents on boll loading of the root system in 2024-2025. BLR, boll loading of the root system; CK, manual topping; X1, zifengding agent; X2, paclobutrazol-copper complex; X3, paclobutrazol-zinc complex. Different lowercase letters indicate significant differences between treatments at the 5% probability level (*p*< 0.05).

The BLR showed an increasing continuously trend as DAT increased (**Figure 5**). The BCR of all chemical topping treatments were higher than those of CK treatment in 28 DAT, the X3 treatment achieved the highest BLR value, compared with CK treatment, the X1, X2, and X3 treatments increased the BLR by 3.30%-3.80%, 9.80%-10.20%, and 12.40%-14.10%, respectively, in 2024 - 2025. However, no statistically significant difference was observed between X2 and X3 treatments in two years.

### 3.4 Cotton biomass

**Figure 6:**
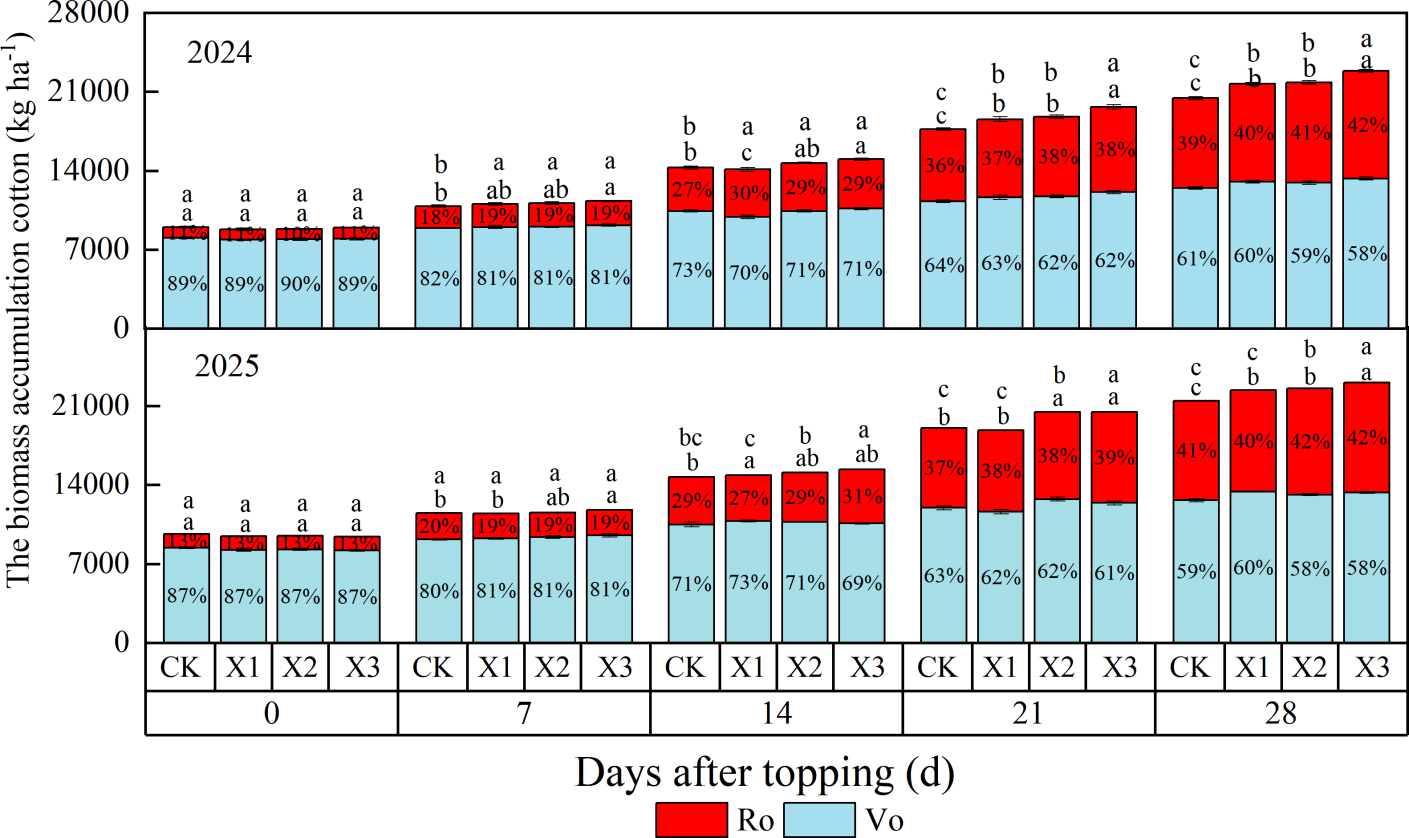
Effects of different chemical topping agents on the biomass accumulation of cotton from 2024-2025. Vo, vegetative organs; Ro, reproductive organs; CK, manual topping; X1, zifengding agent; X2, paclobutrazol-copper complex; X3, paclobutrazol-zinc complex.

Significant differences in cotton biomass were observed among the different chemical topping agents at 28 DAT (**Figure 6**). The X3 treatment achieved the highest dry matter accumulation in 2024 and 2025, from 22,796.41-23,098.58 kg ha^-1^, respectively. Compared with CK treatment, the biomass under X3 treatment increased by 7.62%-10.74%. Furthermore, which was 3.03%-4.21% and 2.24%-5.00% higher than X1 and X2 treatments, respectively. For Vo, the X3 treatment had the highest value. Compared with the CK treatment, the X1, X2, and X3 treatments increased Vo by 10.80%-19.90%, 2.20%-9.10%, and 7.10%-11.50% in 2024-2025, respectively.

### 3.5 Seed cotton yield

The experimental results showed that different chemical topping agents significantly affected cotton yield and yield components (**Table 2**). Compared with CK treatment, the number of bolls per plant under X3 treatment increased by 9.70%-17.00% across the two years. Compared with CK treatment, X1, X2, and X3 treatments were significantly decreased the boll weight by 3.33%, 3.91%, and 3.33% in 2025, respectively. Seed cotton yield under the X3 treatment was 10.44%-12.17% higher than that of CK treatment, with the overall ranking of seed cotton yield across the two years being X3 > X2 > X1 > CK. Lint percentage was reduced under all chemical topping treatments compared with CK treatment. Nevertheless, lint yield under the X3 treatment reached 2342.02-2537.47 kg ha^-1^, withing an increase of 9.20%-11.00% relative to CK treatment. Overall, chemical topping agents promoted synchronized increases in seed cotton and lint cotton yields primarily by increasing the number of bolls per plant, with X3 treatment showing the greatest effectiveness in optimizing reproductive growth and yield formation in cotton.

**Table 2:** The effects of different chemical topping agents on cotton yield and its components during 2024 - 2025.

Year	Treatment	Boll numbers per plant (No)	Boll weight (g)	Seed cotton yield (kg ha^-1^)	Lint percentage (%)	Lint yield (kg ha^-1^)
2024	CK	4.54 c	6.65 a	5080.39 c	42.20 a	2143.91 c
	X1	4.81 b	6.35 ab	5136.62 c	40.83 b	2155.56 c
	X2	4.83 b	6.29 b	5474.71 b	41.97 ab	2235.36 b
	X3	4.98 a	6.43 ab	5610.80 a	41.74 ab	2342.02 a
2025	CK	4.24 b	6.91 a	5249.43 c	43.57 a	2286.31 c
	X1	4.70 a	6.68 b	5414.44 bc	42.69 c	2311.49 bc
	X2	4.87 a	6.64 b	5523.30 b	42.85 bc	2365.08 b
	X3	4.96 a	6.68 b	5888.36 a	43.10 b	2537.47 a

Data are showed in mean value (n=3). CK, manual topping; X1, zifengding agent; X2, paclobutrazol-copper complex; X3, paclobutrazol-zinc complex. Different lowercase letters indicate significant differences between treatments at the 5% probability level (*p*< 0.05).

### 3.6 Fiber quality

Different chemical topping agents exerted no significant overall effects on cotton fiber quality indices, with minimal variations observed across all treatments (**Table 3**). However, STR exhibited certain differences in both years. The X3 treatment consistently achieved the highest STR values in two years, ranging from 32.34 to 34.00 g tex^-1^, which were significantly higher than those of the CK treatment by 3.62%-4.71%. These results indicated that the X3 treatment could effectively enhance cotton fiber breaking strength and thus significantly improve cotton fiber quality. Compared with CK treatment, the chemical topping agents optimized cotton growth and yield without adversely affecting fiber quality, ensuring the consistency of fiber properties.

**Table 3:** The effects of different chemical topping agents on cotton fiber quality during 2024-2025.

Year	Treatment	MIC	UHML (mm)	UNI (%)	STR (g tex^-1^)	SFI (%)
2024	CK	4.35 a	28.65 a	84.98 a	31.21 b	8.18 a
	X1	4.36 a	29.04 a	84.69 a	31.66 ab	8.51 a
	X2	4.61 a	29.23 a	85.17 a	32.29 a	7.92 a
	X3	4.68 a	29.27 a	85.72 a	32.34 a	8.15 a
2025	CK	4.59 a	30.18 a	86.57 a	32.47 c	6.67 a
	X1	4.70 a	30.34 a	86.37 a	32.70 bc	6.60 a
	X2	4.74 a	30.35 a	86.80 a	33.15 ab	6.63 a
	X3	4.86 a	30.42 a	86.70 a	34.00 a	6.57 a

UHML, upper half mean length; UNI, uniformity index; MIC, micronaire; STR, fiber strength; SFI, elongation rate. CK, manual topping; X1, zifengding agent; X2, paclobutrazol-copper complex; X3, paclobutrazol-zinc complex. Different lowercase letters indicate significant differences between treatments at the 5% probability level (*p*< 0.05).

### 3.7 Correlation analysis

**Figure 7:**
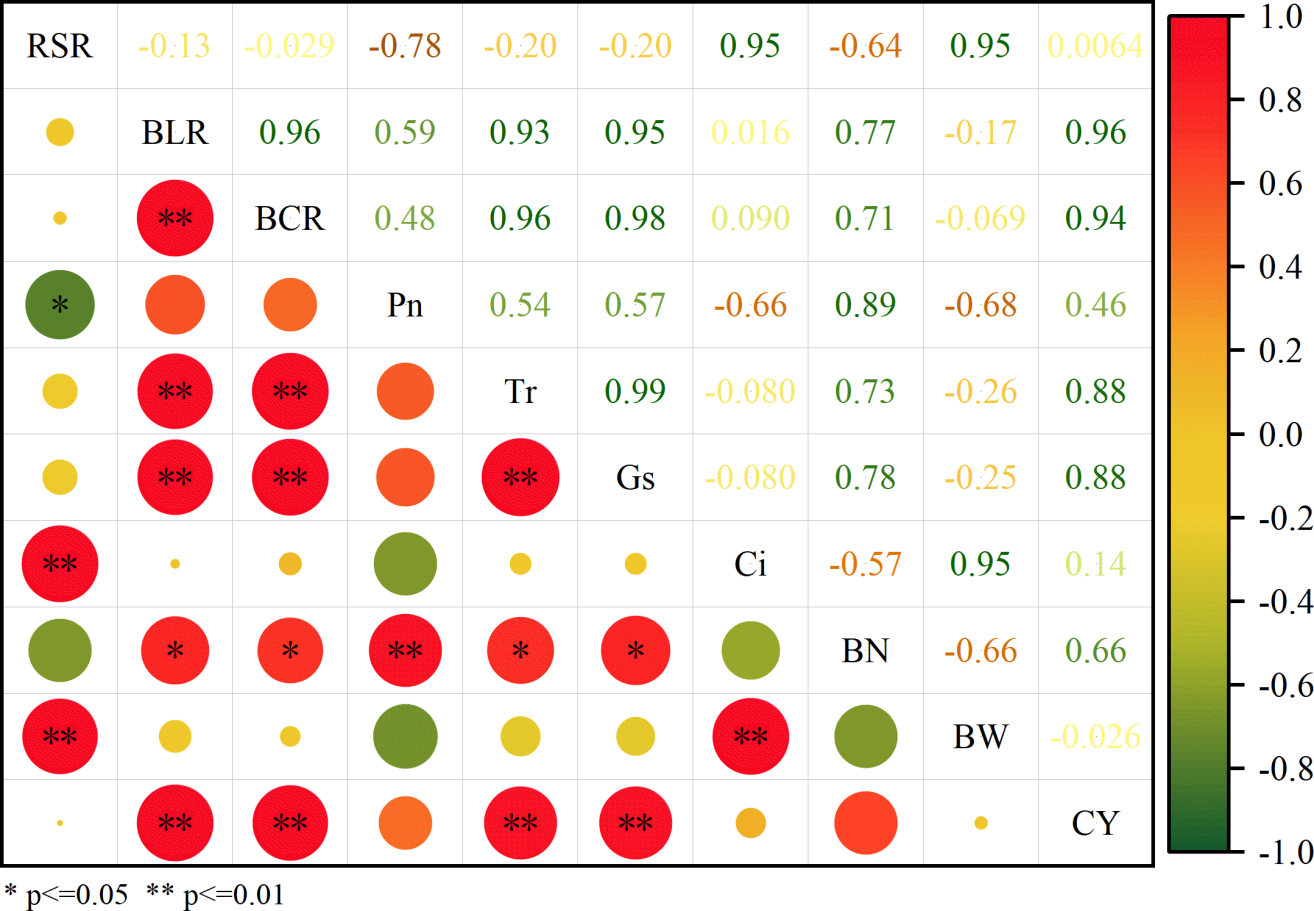
Correlation analysis of root-shoot ratio, photosynthetic parameters, and yield traits in cotton from 2024-2025. RSR, root-shoot ratio; BLR, boll loading of the root system; BCR, boll capacity of the root system; Pn, net photosynthetic rate; Tr, transpiration rate; Ci, Intercellular CO_2_ concentration; Gs, stomatal conductance; BN, boll numbers per plant; BW, boll weight; CY, seed cotton yield.

The results of the correlation analysis between cotton yield and various indicators are presented (**Figure 7**). A significant positive correlation between BCR and BLR. CY was significantly positively correlated with BCR, BLR, Pn, Gs, and Tr, but negatively correlated with Ci. Furthermore, CY was strongly associated with BN, whereas it showed no significant correlation with RSR.

### 3.8 Relationships among cotton growth, photosynthetic parameters, root productivity, biomass, and yield

**Figure 8:**
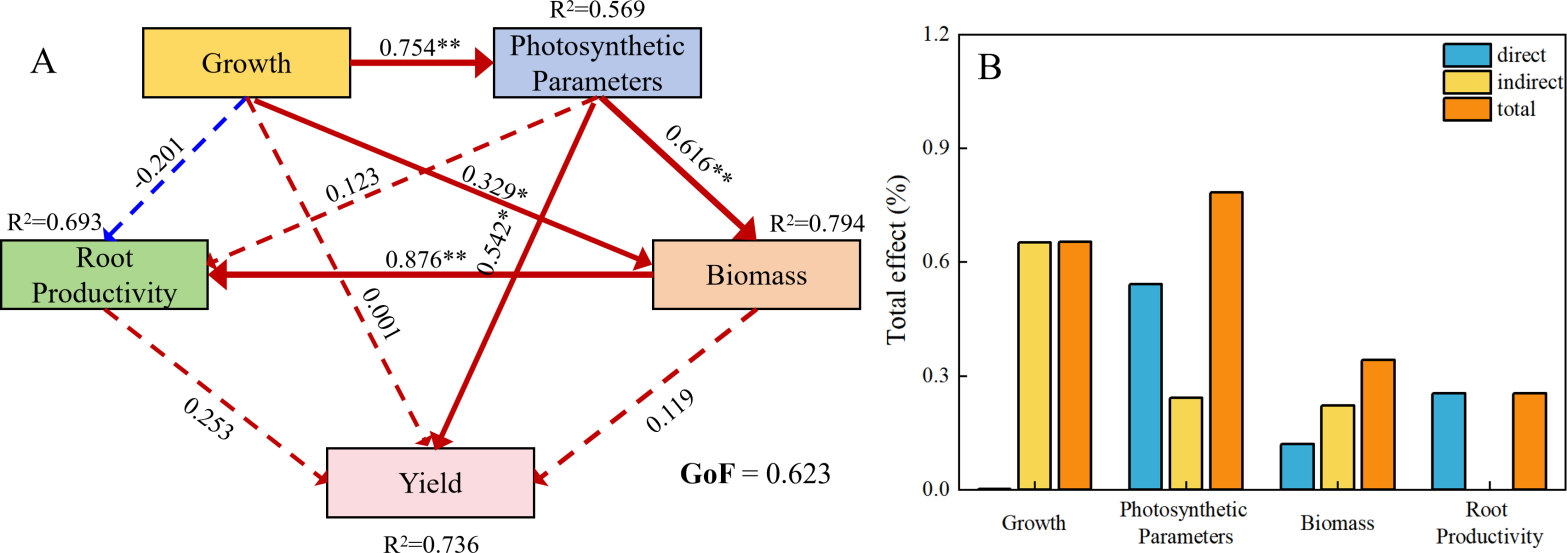
Relationships among plant growth and development, photosynthetic rate, root productivity, dry matter accumulation, and cotton yield estimated by PLS-SEM, partial least squares structural equation modeling **(A)**. And the relative importance of these indexes on cotton seed cotton yield **(B)**. R^2^ represents the amount of variation explained by all paths. A solid-line path indicates that the effects were significant, and a dashed-line path indicates that the effects were not significant. The width of the line the strength of the causal relationship. The red-line path indicates that the effects were positive, and the blue-line path indicates that the effects were negative. *, P< 0.05; **, P< 0.01.

As revealed by the partial least squares structural equation model (PLS-SEM) analysis (**Figure 8**), the formation of cotton yield is primarily directly influenced by photosynthetic parameters, with photosynthetic parameters exerting the most significant total effect on yield. The direct effect of biomass on yield is relatively weak, but it produces a significant indirect effect through the mediation of root system productivity. Root system productivity has a positive direct effect on yield, though its effect magnitude is lower than that of photosynthetic parameters. In contrast, the direct effect of growth on yield is not significant; instead, growth mainly exerts an indirect effect on yield via photosynthetic parameters and biomass. These results indicate that the coordinated improvement of growth processes, biomass, and photosynthetic parameters represents a key regulatory pathway for enhancing cotton yield.

## 4 Discussion

During cotton growth and development, vegetative growth and reproductive growth often occur simultaneously (Oosterhuis, 1990; Chalise et al., 2022). Excessive vegetative growth can inhibit reproductive growth, thereby affecting cotton photosynthesis and dry matter accumulation. This imbalance between vegetative and reproductive growth has long been one of the major bottlenecks limiting high cotton yields (Liu et al., 2025). Therefore, the timely regulation of growth and development processes is of great significance. In traditional cotton production, manual topping has been widely used to inhibit apical dominance. However, this method is associated with drawbacks such as high labor intensity, excessive production costs, and poor operational consistency, making it difficult to meet the demands of modern cotton production (Chen et al., 2025). With the transformation of production modes, chemical topping agents are now widely used in cotton cultivation (Dai et al., 2022). However, the extensive application pattern of a single universal chemical topping agent often results in suboptimal topping efficacy in cotton. Screening chemical topping agents compatible with the local cultivation patterns of Southern Xinjiang has gradually become an indispensable key management technology in current cotton production. The results of this study demonstrated that different chemical topping agents exhibited significant differences in regulating cotton growth and development across two consecutive years of field trials. All chemical topping treatments effectively controlled plant height without regrowth and increased the number of fruit branches. This is consistent with previous findings that mepiquat chloride and triazole-based regulators can inhibit gibberellin synthesis, thereby controlling plant height and promoting the formation of effective fruiting branches (Dai et al., 2022). However, the magnitude of regulatory effects on cotton growth and development varied among different chemical topping agents, which may be attributed to differences in photosynthate partitioning strategies and biomass accumulation rhythms in cotton plants following the application of distinct chemical topping agents.

Photosynthetic capacity serves as a crucial physiological basis determining cotton yield and adaptability (Qin et al., 2023). Chemical topping, which replaces manual topping with exogenous chemical topping agents, inhibits the apical growth dominance of cotton, regulates plant canopy structure and source-sink distribution relationships, and thereby exerts a significant impact on photosynthesis. After the application of chemical topping agents, the apical growth of cotton plants is suppressed, and the proportion of photosynthates allocated to reproductive organs (bolls) increases (Shi et al., 2024). The source-sink feedback regulation mechanism promotes an increase in stomatal conductance of functional leaves and a significant enhancement in net photosynthetic rate. Meanwhile, a moderate increase in transpiration rate helps reduce leaf temperature and avoid photoinhibition of the photosynthetic apparatus under high light intensity. This effect is particularly pronounced during the cotton flowering and boll stage (Tian et al., 2022), extending the photosynthetic functional period of functional leaves by 7–10 days, which is consistent with the results of our study. Compared with manual topping, X3 treatment significantly increased Pn, Gs, and Tr, and exerted an influence on intercellular CO_2_ concentration (**Figure 3**). In addition, at 28 days after topping, the X3 treatment significantly increased the biomass of reproductive organs by 10.82%-18.95% compared with manual topping treatment, and by 8.46%-9.81% and 3.47%-7.47% compared with the X1 and X2 treatments, respectively. This result indicates that an appropriate chemical topping agent can improve CO_2_ diffusion capacity and stomatal regulatory function, thereby promoting the development of reproductive organs during critical growth periods. Relevant studies have pointed out that cotton often exhibits gas diffusion limitation under growth regulation conditions, and enhancing Pn while maintaining reasonable stomatal behavior is usually associated with higher material production efficiency (Gao et al., 2019; Zhang et al., 2024).

As the core organ for cotton to absorb water and nutrients (Chen et al., 2018), the root system directly affects the growth and development of the aboveground parts (Chen et al., 2020), and root productivity serves as the foundation supporting photosynthetic efficiency. In addition, the root system is an important hormone-synthesizing organ besides the apex. An appropriate chemical topping agent can promote the synthesis of cytokinin in roots. Once transported upward to the aboveground parts, cytokinin can delay the senescence of functional leaves and maintain photosynthetic efficiency (Wu et al., 2024). Meanwhile, gibberellin synthesized in roots can regulate the elongation of aboveground fruit branches and coordinate source-sink relationships (Wang et al., 2019). When the root-shoot ratio is abnormally high, growth redundancy is prone to occur between the root system and aboveground parts, leading to an imbalance in the ratio between vegetative and reproductive organs and impairing growth coordination. Our results demonstrated that compared with manual topping, chemical topping treatments significantly reduced the root-shoot ratio, increased the boll loading of the root system and boll capacity of the root system (**Figures 3**-**5**), with the X3 treatment achieving the highest values (0.53-0.55 boll g^-1^ and 3.24-3.46 g g^-1^, respectively). This finding indicates that a suitable chemical topping agent can not only enhance cotton photosynthesis but also promote root development and improve root productivity, thereby constructing an efficient root-shoot synergistic system (Cordeiro et al., 2021; Wu et al., 2019).

Cotton yield formation depends on the synergistic interaction between photosynthetic capacity and biomass partitioning (Nie et al., 2021). Chemical topping inhibits apical dominance and regulates the balance between vegetative and reproductive growth, thereby creating favorable conditions for yield formation (Hayat et al., 2025). Previous studies have indicated that chemical topping generally results in significant yield increases compared to non-topping, whereas compared to manual topping, it typically results in a slight increase or no significant difference (Zhu et al., 2022; Qi et al., 2023). However, the results of this study demonstrate that compared to manual topping, the X3 treatment significantly increased seed cotton yield by 10.44% and 12.17% over the two years, respectively. This yield advantage was primarily attributed to an increase in boll number per plant (**Table 2**) and a higher proportion of biomass allocated to reproductive organs (**Figure 6**). These findings are consistent with the aforementioned trends of enhanced photosynthetic capacity and altered biomass partitioning toward reproductive organs, suggesting that the appropriate chemical topping agent promotes yield formation through a synergistic regulatory pathway of photosynthetic enhancement coupled with preferential reproductive growth.

Previous studies have indicated that chemical regulation measures have no significant effects on cotton fiber quality-related indices (Wu et al., 2025; Zhu et al., 2022). This phenomenon is consistent with the conclusions of multiple studies: the impacts of mepiquat chloride application on quality indices such as micronaire, upper half mean length, and fiber breaking strength are often jointly constrained by ecological conditions and regulation intensity (Ren et al., 2013; Cheema et al., 2009). The results of this study showed that suitable chemical topping agent treatments significantly improved fiber strength without affecting other fiber quality indices (**Table 3**), demonstrating that an appropriate chemical topping agent can not only maintain the stability of fiber quality but also further enhance fiber strength performance. Additionally, chemical topping agents are expected to reduce labor input costs and improve production efficiency, thereby holding potential promotional and application value from the perspective of production practice. As a typical triazole-based growth retardant, paclobutrazol can reduce the growth consumption of ineffective vegetative branches and promote reproductive growth to increase yield by inhibiting gibberellin synthesis and altering source-sink relationships (Desta and Amare, 2021). In this study, the differences in regulatory effects among paclobutrazol-zinc, paclobutrazol-copper, and the zifengding agent may be closely related to the synergistic effects between paclobutrazol and metal ions. As an essential component of various enzyme systems, zinc participates in photosynthetic metabolism and growth and development processes. Previous studies have confirmed that optimizing zinc nutrition levels in cotton can significantly improve growth status and yield performance (Ashraf et al., 2025). Meanwhile, the synergistic regulatory effect of “paclobutrazol-zinc” has also been verified to increase cotton yield (Shen et al., 2025; Sofy et al., 2020). These findings provide theoretical support for the results of this study.

In conclusion, this study systematically investigated the comprehensive effects of different chemical topping agent treatments on cotton growth and development, leaf photosynthetic characteristics, dry matter accumulation and distribution, root productivity, yield, and fiber quality. The results confirmed that the X3 treatment can effectively inhibit apical dominance, enhance leaf photosynthesis, promote rapid dry matter accumulation, improve cotton root productivity, and optimize the root-shoot ratio to construct an efficient root-shoot synergistic system, ultimately increasing cotton yield without compromising fiber quality. This study breaks the extensive “one-size-fits-all” application pattern, realizing the precise and localized adaptation of chemical topping technology. Meanwhile, by deciphering the ecological mechanisms of the agents, this research provides theoretical and technical support for the high-yield, high-quality, efficient, and green cultivation of cotton. Future studies should further integrate molecular biology approaches to explore the response differences of different cotton genotypes to the agents, promoting the development of chemical topping technology toward an intelligent regulation model integrating “cultivar-agent-cultivation”.

## 5 Conclusion

This study demonstrates that chemical topping agents significantly regulate cotton growth and development, photosynthetic performance, root productivity, biomass partitioning, and yield formation. Among all treatments, the X3 treatment achieved the highest seed cotton and lint yields by maintaining high post-topping photosynthetic capacity, improving root boll-bearing capacity and load, and promoting biomass allocation to reproductive organs, without adversely affecting fiber quality. Correlation analysis and partial least squares structural equation modeling (PLS-SEM) further confirmed that photosynthetic parameters are the primary direct drivers of cotton yield formation. Meanwhile, cotton biomass indirectly promotes yield mainly through root productivity. Although root productivity makes a weaker direct contribution to yield than photosynthetic parameters, it serves as a critical mediator that converts biomass accumulation into final yield. These results indicate that chemical topping promotes cotton yield via a synergistic regulatory pathway of “photosynthetic enhancement-root productivity optimization-efficient shift from vegetative to reproductive growth”. Overall, the X3 treatment represents an efficient and sustainable chemical topping agent suitable for cotton region south of the Tianshan Mountains in Xinjiang, providing theoretical and technical support for high-yield, high-quality, efficient, and green cotton production.

## Data Availability

The original contributions presented in the study are included in the article/supplementary material. Further inquiries can be directed to the corresponding authors.
